# Role of Radical Cystectomy in Metastatic Bladder Cancer

**DOI:** 10.3390/jcm15218586

**Published:** 2026-11-04

**Authors:** Jonathan Wong, Ryan Wong, Zachary Hamilton, Luke Wang

**Affiliations:** 1 School of Medicine, Saint Louis University, St. Louis, MO 63104, USA; jonathan.wong@health.slu.edu; 2 Division of Urology, SSM Health Saint Louis University, St. Louis, MO 63110, USA

**Keywords:** metastatic bladder cancer, radical cystectomy, treatment utilization, health disparities

## Abstract

**Background/Objectives:** The role of radical cystectomy (RC) in clinically metastatic (cM+) bladder cancer is unclear. This study aimed to identify national clinical practice patterns, factors associated with receipt of RC, and survival outcomes among patients with cM+ bladder cancer before the immunotherapy and antibody–drug conjugate era. **Methods:** In this retrospective cohort study, 18,360 patients with cM+ bladder cancer were identified from the National Cancer Database between 2006 and 2016. Demographics, comorbidities, cancer stage, and surgical outcomes were compared between patients who received RC and those managed without cystectomy (No RC). Multivariable logistic regression was used to identify factors associated with receiving RC, and overall survival was evaluated with Kaplan–Meier analysis and a multivariable Cox model with time measured from diagnosis, adjusted for age, sex, race, Charlson Comorbidity Index (CCI) score, clinical T stage, and histology. **Results:** Among the 18,360 patients with cM+ bladder cancer, 987 (5.4%) underwent RC. Patients who underwent RC were significantly younger, more likely to be White, and had lower CCI scores. Utilization of RC remained stable throughout the study period. On multivariable logistic regression, increasing age, Black race, and CCI score ≥ 3 were associated with lower odds of receiving RC, whereas cT3/4 disease and urothelial histology were predictors of receiving RC. Kaplan–Meier analysis demonstrated higher overall survival in the RC group. Postoperative mortality was 4.6% at 30 days and 23.6% at 90 days. **Conclusions:** Radical cystectomy was infrequently performed among patients with cM+ bladder cancer and was concentrated among younger, healthier patients. The survival difference between groups reflects selection and cannot be interpreted as a causal survival benefit from RC, since surgical intent, metastatic burden, and postoperative disease status are unavailable. Our findings provide a descriptive, population-level reference for comparison with future studies of RC in the current treatment era.

## 1. Introduction

Bladder cancer is one of the most common cancers globally, with an estimated 600,000 new cases diagnosed each year worldwide and approximately 80,000 new diagnoses annually in the United States [1]. Notably, about 5–6% of patients present with clinically metastatic (cM+) or Stage IV bladder cancer at the time of diagnosis, which is associated with poor survival compared to earlier-stage disease [2]. Platinum-based chemotherapy has long been used as the first-line systemic treatment for managing cM+ bladder cancer. However, the treatment paradigm has evolved in recent years with the addition of novel immunotherapy agents. The introduction of these agents has improved survival among patients and has altered the treatment landscape since 2016. The role of surgery in the setting of metastatic disease remains limited.

From a surgical perspective, radical cystectomy (RC) is regarded as a standard curative treatment for local advanced bladder cancer [3]. In contrast, for patients with cM+ bladder cancer, the role of RC is less clearly defined. RC may be considered for palliative intent to alleviate severe local symptoms such as hematuria, pelvic pain, or urinary obstruction that may not be controlled with systemic therapy [4,5]. Additionally, RC may be performed with cytoreductive intent or as part of a multimodal treatment in highly selected patients, such as those with limited metastatic burden or a favorable response to systemic therapy [5,6,7]. Despite its potential benefits, the utilization of RC in metastatic settings remains an area of uncertainty, largely due to the associated significant perioperative risks. Current data on this procedure are limited; thus, clinical patterns of patients and selection criteria remain unclear. Therefore, our study aimed to use a large national dataset to examine the utilization patterns, patient selection factors, and survival outcomes associated with RC in the cM+ bladder cancer population. We hypothesized that utilization would remain low and observed survival would remain poor.

## 2. Materials and Methods

The National Cancer Database (NCDB) was retrospectively reviewed for patients diagnosed with cM+ bladder cancer who underwent radical cystectomy (RC) or did not undergo radical cystectomy (No RC) from 2006–2016. The NCDB is a joint quality improvement project produced by the American Cancer Society and the American College of Surgeons, which captures data from approximately 30% of United States hospitals and 70% of all patients newly diagnosed with cancer [8]. The data collected are de-identified, and the categories include patient socio-demographics, tumor characteristics, clinical and pathologic staging, definitive treatments, and all-cause mortality. Details of specific systemic regimens and cancer-specific survival are not included. All information is collected in a HIPAA-compliant manner, and data submitted to the NCDB undergo extensive quality monitoring and validity review on an annual basis. The data utilized in this study came from the publicly shared and de-identified NCDB dataset. Approval by the institutional review board was not deemed necessary because no patient or hospital identifiers were analyzed. The American College of Surgeons and the Commission on Cancer have not verified and are not responsible for the analytic or statistical methodology employed or the conclusions drawn from these data by the investigators.

**Figure 1:**
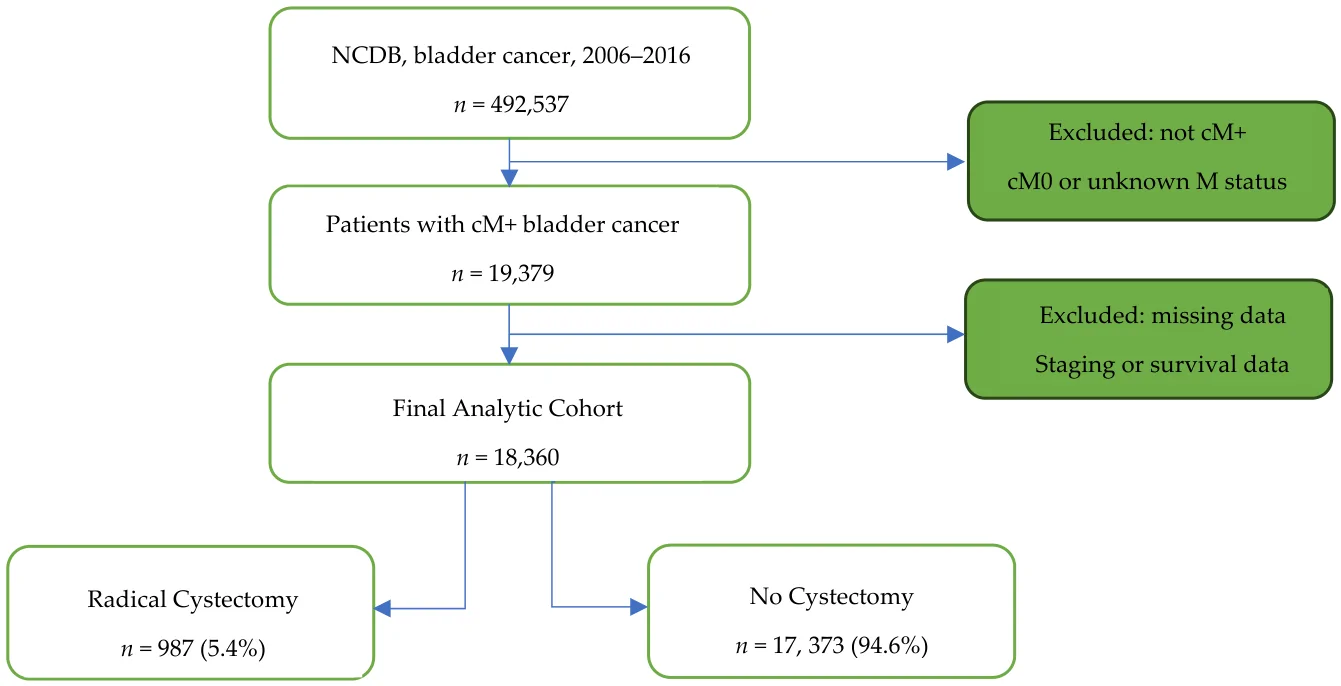
Flow diagram of cohort selection from the National Cancer Database (NCDB), 2006–2016, showing exclusions and the final groups by receipt of radical cystectomy.

Patients were identified using ICD-O-3 primary site codes C67.0–C67.9. All patients with clinically metastatic (cM+), defined as clinical M1 disease using the AJCC staging were included, regardless of cT or cN disease. Patients with regional lymph node involvement without distant metastasis (cN+, M0) were not included. Radical cystectomy was identified using the NCDB surgical procedure of the primary site codes 60–64 and 71, which corresponds to radical cystectomy with or without urinary diversion, anterior exenteration, or cystoprostatectomy. Patients were excluded if they lacked staging information, surgical outcomes, or survival data (Figure 1). The following data were extracted: age, sex, race, insurance coverage, income status, Charlson Comorbidity Index (CCI) score (0–3+), clinical T stage, histology, all-cause mortality, and length of follow-up. Follow-up time was defined as the interval from the date of diagnosis to death or last follow-up, as predefined in the NCDB. Histology was recorded using ICD-O-3 histology codes and classified as urothelial or variant, with variant histology defined as any histology other than urothelial carcinoma, including adenocarcinoma, carcinosarcoma, large cell neuroendocrine carcinoma, micropapillary carcinoma, neuroendocrine carcinoma not otherwise specified, sarcomatoid carcinoma, small cell carcinoma, and squamous cell carcinoma. Treatment facilities that accrued 500 or more newly diagnosed cancer cases per year were considered high-volume (including academic centers and comprehensive cancer centers), while facilities that accrued fewer than 500 new cases per year were labeled low volume, per NCDB stratification [9]. Facility type was not included in the multivariable model. The NCDB does not record the indication for surgery; therefore, RC could not be classified by intent. Surgical outcomes included margin status, utilization of lymph node dissection, robotic-assisted approach, 30-day complications requiring readmission, and 30-day and 90-day mortality. Thirty- and 90-day postoperative mortality were defined as death from any cause within 30 and 90 days after RC, respectively, among the 987 patients. The primary outcome was utilization of radical cystectomy and associated clinical practice patterns. The secondary outcomes were overall survival and complication rates.

The analysis period was 2006–2016. Patients before 2017 were included so that the analysis would reflect a population that had not received novel immunotherapy, allowing more discrete evaluation of surgical and survival outcomes, since novel immunotherapy has substantially improved survival outcomes compared to traditional platinum-based chemotherapy [10].

Student’s *t*-test was performed for continuous variables, and Fisher’s exact test or the Pearson chi-square test was used for categorical variables. Analyses were performed comparing RC cohorts. Linear regression analysis was used to determine the rate of RC utilization over time. A multivariable logistic regression analysis evaluating variables associated with RC utilization included variables that were statistically significant on univariate analysis, including patient demographics, CCI score, and clinical tumor stage. The yearly utilization analysis was restricted to patients with complete data. Overall survival was measured in months from the date of diagnosis to death or last follow-up, as recorded in the NCDB, and survival curves for the RC and No RC groups were estimated with the Kaplan–Meier method and compared with the log-rank test. Patients with inconsistent follow-up time entries were excluded from the Kaplan–Meier analysis. One- and 2-year survival, median survival, and the numbers of patients at risk at 0, 12, and 24 months are reported. To account for differences between the groups, we fitted a multivariable Cox proportional hazards model for death that included receipt of RC, age, sex, race, CCI score, clinical T stage, and histology. These covariates were chosen since they potentially influence clinician treatment decisions and survival and are available data in the NCDB [4,11]. Receipt of RC was modeled as a fixed exposure, with time measured from the date of diagnosis. The proportional hazards assumption was assessed and met for all covariates. Analyses were performed using SPSS v27 (IBM Corp., Armonk, NY, USA), with a *p*-value < 0.05 denoting statistical significance.

## 3. Results

### 3.1. Patient Demographics and Clinical Characteristics

Demographic data, clinical tumor features, and comorbidity profiles of cM+ patients, stratified by receipt of RC, are summarized in Table 1. Among the 18,360 patients included in the cohort, the vast majority (94.6%) did not undergo cystectomy, while a smaller subset (987 patients, 5.4%) underwent RC.

Patients receiving RC were significantly younger, with a mean age of 66.4 years compared with 71.0 years in the No RC group (*p* < 0.001). Among all patients with cM+ bladder cancer, 86.8% identified as White. RC was performed in 5.5% of White patients, compared with 4.3% of Black patients and 4.7% of patients classified as non-White or non-Black. Men represented 69.8% of the overall cohort. Patients with lower CCI scores were more likely to undergo RC, and patients who underwent RC had higher proportions of cT3 (14.0% vs. 6.8%) and cT4 (24.7% vs. 18.0%) stages compared with the No RC group. Insurance status was similar between the groups, whereas income status differed (*p* = 0.028). Urothelial histology was slightly more common in the RC group (80.9% vs. 76.1%).

### 3.2. Perioperative Outcomes

Perioperative and survival outcomes stratified by RC status are summarized in Table 2. Among patients undergoing RC, 8.5% were readmitted within 30 days, 29% did not receive lymphadenectomy, 30% had positive margins, and 9% underwent a robotic approach. The mean time from diagnosis to RC was 82.5 ± 78.8 days. Length of follow-up was significantly longer among patients in the RC group compared with the No RC group (17.3 vs. 9.6 months, *p* < 0.001). Overall mortality was high among cM+ bladder cancer patients (80.9% in No RC; 79.1% in RC). These values represent the proportion of patients who died by the end of follow-up and do not account for the timing of death or differences during the follow-up duration. Therefore, these values do not conflict with the difference in median overall survival. Postoperative mortality after RC was 4.6% within 30 days and 23.6% within 90 days.

### 3.3. Trends and Predictors in Utilization of Radical Cystectomy

**Figure 2:**
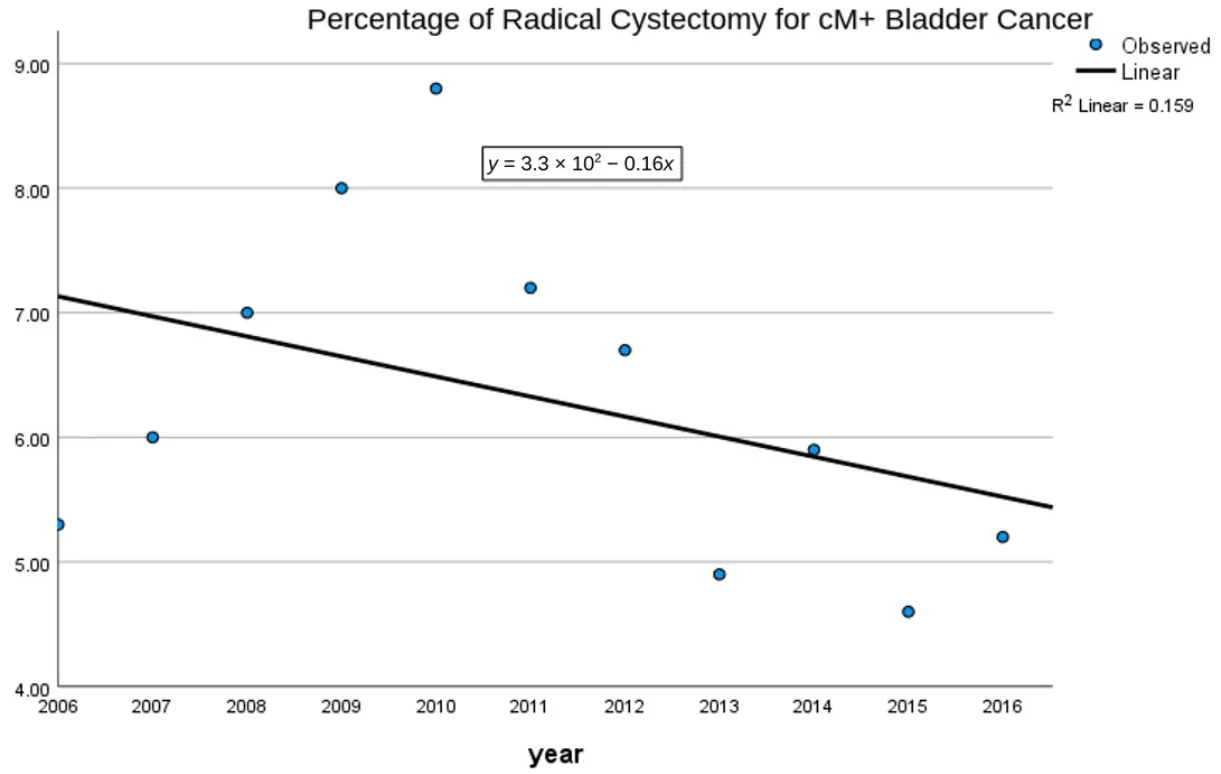
Yearly utilization of radical cystectomy among patients with cM+ bladder cancer, 2006–2016. Percentages were calculated among patients with complete data (*n* = 14,192).

Yearly utilization was analyzed among the 14,192 patients with complete data (879 who underwent RC). Linear regression analysis indicated that utilization of RC remained stable and unchanged during the study period (R^2^ = 0.159, *p* = 0.224; Table 3, Figure 2). Table 4 summarizes factors associated with receiving RC on multivariable logistic regression analysis. Older age (*p* < 0.001), Black race (*p* = 0.001), and CCI score ≥ 3 (*p* < 0.001) were significantly associated with a lower likelihood of receiving RC. In contrast, higher cT stage was significantly associated with receiving RC, notably cT3 and cT4 disease, with odds ratios of 3.401 and 2.221, respectively (both *p* < 0.001). Additionally, urothelial histology was associated with a higher likelihood of receiving RC (OR 1.420, *p* < 0.001).

### 3.4. Overall Survival

**Figure 3:**
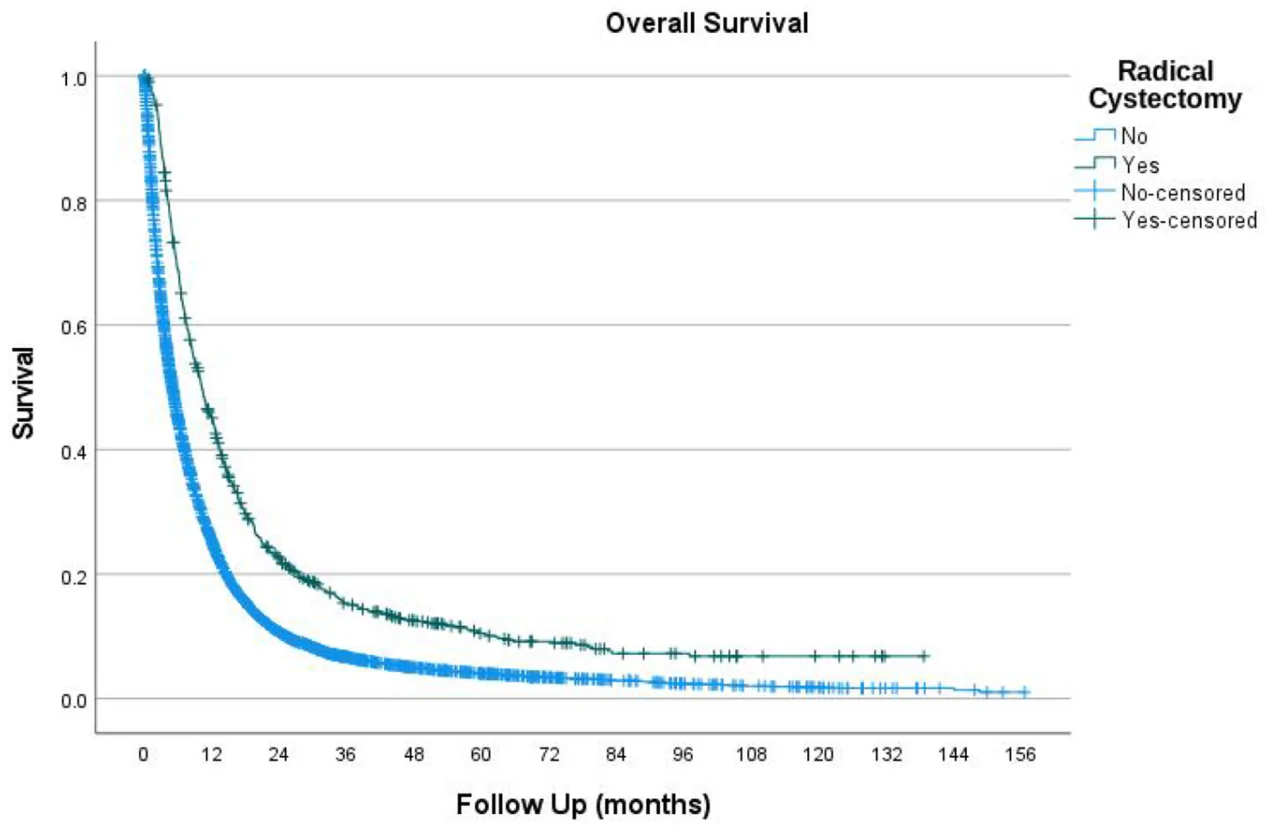
Kaplan–Meier curves of overall survival, measured from the date of diagnosis, among patients with cM+ bladder cancer in the NCDB (2006–2016), stratified by receipt of RC. Medial survival was 10.2 months with RC and 4.9 months without RC (log-rank *p* < 0.001).

Kaplan–Meier survival analysis for patients treated with RC compared with those not treated with RC is shown in Figure 3. The RC group demonstrated higher overall survival, with a median survival time of 10.2 months compared with 4.9 months in the No RC group (log-rank *p* < 0.001). One-year overall survival was 45.1% in the RC group and 25.3% in the No RC group, and 2-year overall survival was 22.5% and 10.7%, respectively. Kaplan–Meier estimates of overall survival and the numbers of patients at risk are provided in Table 5 and Table 6, respectively. The Kaplan–Meier analysis included 16,204 patients (15,298 No RC and 906 RC) after excluding 2156 patients with inconsistent follow-up time entries.

Multivariable Cox analysis in Table 7 showed receipt of RC was associated with a lower hazard of death (HR 0.632, 95% CI 0.588–0.680; *p* < 0.001) after adjusting for age, sex, race, CCI score, clinical T stage, and histology. Patients of older age (HR 1.014), higher CCI score (HR 1.164, 1.355, and 1.551 for scores of 1, 2, and 3+ respectively), and higher clinical T stage were associated with higher hazard of death. Male sex (HR 0.927) and urothelial histology (HR 0.802) were associated with a lower hazard. Black race was not significantly associated with survival (HR 1.049, 95% CI 0.994–1.108; *p* = 0.082).

## 4. Discussion

In this analysis of NCDB cM+ bladder cancer patients in the pre-immunotherapy era from 2006 to 2016, 5.4% of patients underwent RC, and this utilization did not increase over time. Younger patients with lower CCI scores were more likely to undergo RC, and those who did undergo RC experienced a median survival of 10.2 months.

Large, invasive tumors can produce increasingly severe symptoms, such as hematuria, pelvic pain, or urinary obstruction, due to tumor-related obstruction of normal urinary flow [5]. These symptoms may prompt consideration of RC for symptomatic relief when nonsurgical management (immunotherapy, radiotherapy, or chemotherapy) is insufficient [12,13]. RC may also be performed with cytoreductive intent, with the aim of reducing primary tumor burden and potentially enhancing the effectiveness of systemic therapy [5,6]. Additionally, RC may be a component of a multimodal plan in highly selected patients, such as those with limited metastatic disease or a favorable response to systemic treatment [7]. There is selection bias in the use of RC, given that patients who underwent RC were younger and had fewer comorbidities, suggesting that RC was offered to carefully selected patients with cM+ bladder cancer [4,11]. The NCDB does not record the surgical indication or intent for RC; thus, we cannot determine the reasons for these patterns. The median survival was longer in the RC group (10.2 vs. 4.9 months), and receipt of RC remained associated with a lower hazard of death after adjusting for age, sex, race, comorbidity, clinical T stage, and histology. In this model, we found older age, higher comorbidity burden, and higher clinical T stage were associated with survival. However, this adjusted association should not be interpreted as a survival benefit of RC since the NCDB lacks performance status, response to systemic therapy, and metastatic burden. Additionally, because survival time was measured from diagnosis and RC was modeled as a fixed exposure, the adjusted hazard ratio remains affected by immortal-time bias, which favors patients who survived long enough to undergo RC.

Postoperative mortality was substantial in our cohort, at 4.6% within 30 days and 23.6% within 90 days of surgery. Although 30-day mortality is a widely used metric as a measure of postoperative risk and surgical outcomes, this window underestimates the early mortality of RC in patients with cM+ bladder cancer [14,15]. In our cohort, 90-day mortality was approximately five times higher than 30-day mortality, indicating that assessment limited to 30 days would fail to capture a large proportion of early deaths. However, 90-day mortality likely reflects death from progressive metastatic disease, deaths related to perioperative complications, or deaths from other comorbid or unrelated causes. The NCDB does not record cause of death; thus, the contribution to these factors cannot be determined. Furthermore, when RC is performed for symptom control, the potential for improvement in quality of life must be weighed against substantial early mortality, 8.5% 30-day readmission rate, and limited median overall survival of 10.2 months. When RC is performed with cytoreductive or multimodal intent with the purpose of prolonging survival, the observed survival difference cannot be attributed to RC because the NCDB lacks several clinical variables that influence treatment selection and prognosis. These mortality estimates are best interpreted as descriptive benchmarks of early postoperative risk observed in our cohort.

The factors associated with receipt of RC in our cohort are consistent with those reported for patients undergoing RC with curative intent. Gore et al. and Williams et al. similarly identified older age, greater comorbidity burden, and race as independent predictors of a reduced likelihood of undergoing RC among patients with non-metastatic muscle-invasive disease [16,17]. Our findings suggest similar patterns in the metastatic setting, with increasing age, higher CCI scores, and Black race associated with lower odds of receiving RC, suggesting that patient characteristics linked to selection for surgery in localized disease may also be associated with selection in cM+ bladder cancer. These observed patterns should be interpreted as descriptive associations, since the decision to operate may have depended on other factors that were not captured, such as performance status, symptom burden, the extent and sites of metastatic disease, response to systemic therapy, patient or surgeon preferences, and institutional practices [4,5].

Systemic therapy remains the mainstay of treatment for cM+ urothelial carcinoma, with PD-1/PD-L1-targeted immune checkpoint inhibitors incorporated into standard care since 2016. Recently, the combination of pembrolizumab, a PD-1 inhibitor, and enfortumab vedotin, an antibody–drug conjugate, has yielded improved median overall survival compared with platinum-based chemotherapy and gemcitabine (31.5 vs. 16.1 months) and is now a first-line treatment option for cM+ urothelial cancer [18,19]. Our cohort predates these therapies and therefore provides a historical description of surgical utilization and selection, limiting the applicability of our findings to the current clinical practice. Modern systemic therapy may alter prognosis and influence which patients are considered for surgery. As a result, the patient selection, survival, and perioperative outcomes we describe reflect an earlier treatment era and do not represent outcomes among patients with cM+ bladder cancer managed with contemporary systemic therapy. Future studies will need to evaluate RC in a larger cohort of patients receiving concurrent immunotherapy.

In our cohort, Black patients had significantly lower odds of receiving RC after adjustment for age, comorbidities, clinical stage, income, and histology. Prior studies indicate that although bladder cancer incidence is higher among White populations, Black populations are more likely to present with metastatic or later-stage disease [20,21]. Previous research has also reported that Black populations with bladder cancer are less likely to be treated at high-volume centers, where cystectomy is more often performed [22,23], and that they are more likely to decline potentially curative surgery which has been linked to medical mistrust [24,25]. Differences in referral patterns, clinician-patient communication, and delays in diagnostic evaluation have also been proposed as contributors to this disparity [26]. Differences in tumor characteristics between racial groups have also been described, such as variation in the incidence of more aggressive variant histologic subtypes [27,28]. However, research examining these biologic and tumor-related differences has not shown that they influence treatment selection, whereas previous studies have attributed disparities in bladder cancer care predominantly to social and access-related factors [29]. These explanations were not examined in the present study and are offered as hypotheses from prior literature. Our findings represent an observed association and do not establish a causal inference for the lower rate of RC in Black patients.

Insurance status is one mechanism through which access to care may influence treatment; however, our analysis did not demonstrate a significant difference in the proportion of uninsured patients between those who did and did not undergo RC (3.0% vs. 4.0%, *p* = 0.172). Few patients were uninsured, and the available data do not capture insurance types, adequacy of coverage, and referral patterns. Given the limited dataset, our results do not exclude lack of insurance as a potential barrier to accessing RC. While previous research has suggested that insurance status and other barriers to healthcare access contribute to racial disparities in bladder cancer treatment, future studies with data on access to care, referral pathways, and socioeconomic status are needed to characterize how structural barriers influence receipt of RC in the era of modern systemic therapy [30].

Our study has several important limitations. The retrospective design and use of the NCDB introduce selection bias and unmeasured confounding despite controlling for available variables with multivariable regression analysis. Survival was measured from the date of diagnosis, so patients who underwent RC had to survive a mean of 82.5 days to reach surgery, introducing immortal-time bias in favor of the RC group. Patients selected for RC were also likely fit enough to undergo surgery, which further limits comparison of survival between the groups. Because RC was modeled as a fixed exposure from diagnosis rather than as a time-dependent exposure, and we did not perform a time-dependent or landmark analysis, the adjusted hazard ratio (HR 0.632) remains affected by this bias and cannot establish a survival benefit of RC. Additionally, covariate selection for the multivariable logistic regression analysis was based on univariate statistical significance, which may have excluded clinically relevant confounders that did not reach statistical significance on univariate analysis. The yearly utilization analysis was a complete-case analysis, and patients with missing data were more often in the No RC group, which may introduce selection bias. We were also limited by the information captured within the NCDB, which does not include data on indications for RC (e.g., palliative vs. cytoreductive intent), symptom relief, systemic therapy regimen, preoperative performance status, or postoperative quality of life—all of which are important for understanding surgical selection. The NCDB does not capture metastatic burden with sufficient granularity to account for the number, distribution, or extent of metastatic lesions. Variant histology in the NCDB does not indicate pure variant histology, so patients in the variant category may additionally have urothelial components. The NCDB also lacks information on surgical experience, resource availability, and perioperative care protocols. Finally, the study evaluated patients through 2016, before the widespread adoption of key immunotherapies (e.g., pembrolizumab, atezolizumab, nivolumab) currently used in the treatment of cM+ bladder cancer.

## 5. Conclusions

RC was rarely utilized among patients with cM+ bladder cancer, with an average of 5% of patients undergoing RC from 2006 to 2016. Younger age, lower comorbidity burden, and more advanced local tumor stage were clinical factors associated with a greater likelihood of receiving RC among patients with cM+ bladder cancer, and median survival was longer in the RC group. Nevertheless, postoperative mortality was substantial, and the utilization and role of RC in metastatic disease remain difficult to define. Our findings are limited by the data from NCDB, which does not capture surgical intent, symptom relief, quality of life, or metastatic burden. Therefore, these findings cannot be used to assess the benefit of RC or to guide patient selection. Accordingly, our results should be interpreted as a historical, descriptive baseline for RC selection and not as evidence for or against offering RC in current clinical practice. Future studies in the current immunotherapy era may benefit from examining RC in patients treated with modern systemic therapy, incorporating surgical intent, treatment response, quality-of-life outcomes, and survival.

## Figures and Tables

**Table 1:** Patient demographics and clinical tumor characteristics of metastatic bladder cancer patients.

Variable	All cM+ Bladder (*n* = 18,360)	No Cystectomy (*n* = 17,373)	Cystectomy (*n* = 987)	Significance
Mean Age	70.8 ± 11.9	71.0 ± 11.9	66.4 ± 11.0	<0.001
Race				0.044
White	15,935 (86.8%)	15,053 (86.6%)	882 (89.4%)	
Black	1809 (9.9%)	1733 (10.0%)	76 (7.7%)	
Other	616 (3.4%)	587 (3.4%)	29 (2.9%)	
Male	12,810 (69.8%)	12,118 (69.8%)	692 (70.1%)	0.829
CCI Score				<0.001
0	12,456 (67.8%)	11,745 (67.6%)	711 (72.0%)	
1	3777 (20.6%)	3596 (20.7%)	181 (18.3%)	
2	1428 (7.8%)	1352 (7.8%)	76 (7.7%)	
3+	699 (3.8%)	680 (3.9%)	19 (1.9%)	
cT Stage				<0.001
cT1	2175 (11.8%)	2102 (12.1%)	73 (7.4%)	
cT2	5076 (27.6%)	4841 (27.9%)	235 (23.8%)	
cT3	1325 (7.2%)	1187 (6.8%)	138 (14.0%)	
cT4	3372 (18.4%)	3128 (18.0%)	244 (24.7%)	
cTx	6412 (34.9%)	6115 (35.2%)	297 (30.1%)	
cN+	6075 (33.1%)	5713 (32.9%)	362 (36.7%)	0.015
Income Status				0.028
<$38,000	3333 (18.2%)	3156 (18.2%)	177 (18.0%)	
$38,000–47,999	4474 (24.5%)	4214 (24.4%)	260 (26.5%)	
$48,000–62,999	4886 (26.7%)	4662 (26.9%)	224 (22.8%)	
$63,000+	5587 (30.6%)	5267 (30.4%)	320 (32.6%)	
Uninsured	718 (3.9%)	688 (4.0%)	30 (3.0%)	0.172
Pathology				<0.001
Urothelial	14,014 (76.3%)	13,216 (76.1%)	798 (80.9%)	
Variant	4346 (23.7%)	4157 (23.9%)	189 (19.1%)	

cM+ = metastatic bladder cancer. Variant histology includes adenocarcinoma, carcinosarcoma, large cell neuroendocrine carcinoma, micropapillary carcinoma, neuroendocrine carcinoma not otherwise specified, sarcomatoid carcinoma, small cell carcinoma, and squamous cell carcinoma.

**Table 2:** Perioperative and survival outcomes for metastatic bladder cancer patients, by cystectomy status.

Variable	All cM+ Bladder (*n* = 18,360)	No Cystectomy (*n* = 17,373)	Cystectomy (*n* = 987)	Significance
30-Day Readmission	N/A	N/A	84 (8.5%)	
pN Stage	N/A	N/A		
pN0			295 (29.9%)	
pNx			285 (28.9%)	
pN+			407 (41.2%)	
Positive Margin	N/A	N/A	294 (29.8%)	
Robotic Approach	N/A	N/A	92 (9.3%)	
Time from diagnosis to cystectomy (days)	N/A	N/A	82.5 ± 78.8	
Length of Follow-Up (months)	10.1 ± 15.2	9.6 ± 14.7	17.3 ± 21.3	<0.001
Mortality (all patients)	14,839 (80.8%)	14,058 (80.9%)	781 (79.1%)	0.165
30-Day Postoperative Mortality	N/A	N/A	45 (4.6%)	
90-Day Postoperative Mortality	N/A	N/A	233 (23.6%)	

cM+ = metastatic bladder cancer.

**Table 3:** Yearly utilization of radical cystectomy, 2006–2016.

Year	All cM+	RC	%
2006	766	41	5.3
2007	812	49	6.0
2008	857	60	7.0
2009	1002	80	8.0
2010	1212	107	8.8
2011	1312	95	7.2
2012	1518	102	6.7
2013	1582	78	4.9
2014	1615	95	5.9
2015	1719	79	4.6
2016	1797	93	5.2
Total	14,192	879	6.2

RC = radical cystectomy. Linear regression: R^2^ = 0.159, *p* = 0.224. The yearly counts include only patients with complete data (*n* = 14,192, including 879 who underwent RC). The overall RC rate in the full cohort (*n* = 18,360) was 5.4%.

**Table 4:** Multivariable logistic regression evaluating variables associated with receipt of radical cystectomy.

Variable	OR	95% CI Low	95% CI High	*p*-Value
Age (per 1 year increase)	0.969	0.964	0.975	<0.001
Race (White reference)				
Black	0.661	0.515	0.848	0.001
Other	0.829	0.566	1.216	0.338
CCI Score (0 reference)				
1	0.921	0.777	1.091	0.340
2	1.036	0.810	1.325	0.780
3+	0.531	0.333	0.845	0.008
cT Stage (cT1 reference)				
cT2	1.375	1.050	1.802	0.021
cT3	3.401	2.526	4.579	<0.001
cT4	2.221	1.692	2.915	<0.001
cTx	1.472	1.130	1.918	0.004
cN+	0.965	0.840	1.108	0.615
Income Status (<$38,000 reference)				
$38,000–47,999	1.081	0.884	1.321	0.449
$48,000–62,999	0.844	0.686	1.038	0.109
$63,000+	1.120	0.922	1.361	0.254
Urothelial Cell Histology	1.420	1.202	1.677	<0.001

CI = confidence interval; OR = odds ratio.

**Table 5:** Kaplan–Meier overall survival by receipt of radical cystectomy.

Variable	1-Year OS	SD	2-Year OS	SD	Median OS
No RC	25.3%	0.4	10.7%	0.3	4.9 months
RC	45.1%	1.7	22.5%	1.4	10.2 months

OS = overall survival; SD = standard deviation as reported; RC = radical cystectomy. Log-rank *p* < 0.001.

**Table 6:** Number of patients at risk by follow-up time and receipt of radical cystectomy.

Variable	0 Months	12 Months	24 Months
No RC	15,298	3634	1382
RC	906	394	182

RC = radical cystectomy. Numbers at risk correspond to the Kaplan–Meier curves in Figure 3.

**Table 7:** Multivariable Cox proportional hazards model for death.

Variable	HR	95% CI Low	95% CI High	*p*-Value
Age (per 1 year increase)	1.014	1.012	1.015	<0.001
Male sex	0.927	0.895	0.960	<0.001
Race (White reference)				
Black	1.049	0.994	1.108	0.082
Other	0.922	0.841	1.011	0.086
CCI Score (0 reference)				
1	1.164	1.118	1.212	<0.001
2	1.355	1.274	1.440	<0.001
3+	1.551	1.416	1.700	<0.001
cT Stage (cT1 reference)				
cT2	1.148	1.084	1.216	<0.001
cT3	1.227	1.136	1.325	<0.001
cT4	1.319	1.241	1.402	<0.001
cTx	1.315	1.244	1.390	<0.001
Radical cystectomy	0.632	0.588	0.680	<0.001
Urothelial Cell Histology	0.802	0.772	0.834	<0.001

CI = confidence interval; HR = hazard ratio.

## Data Availability

The data used in this study are derived from the NCDB.
